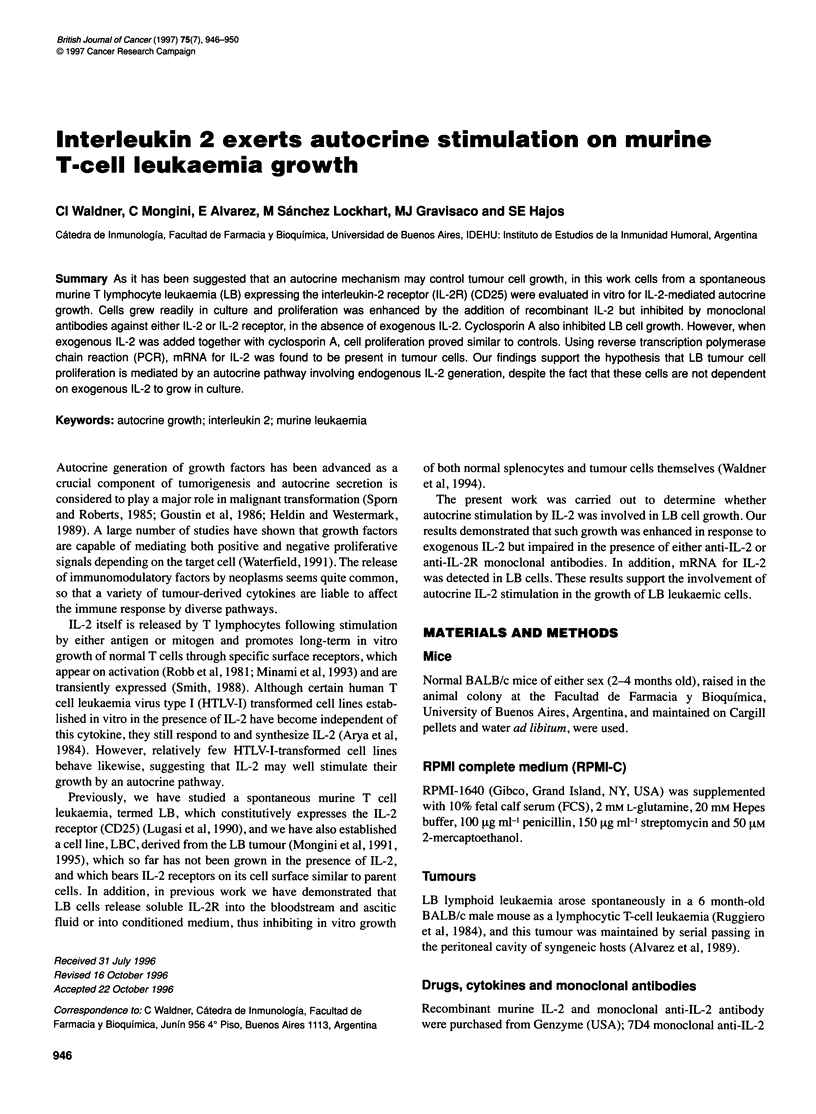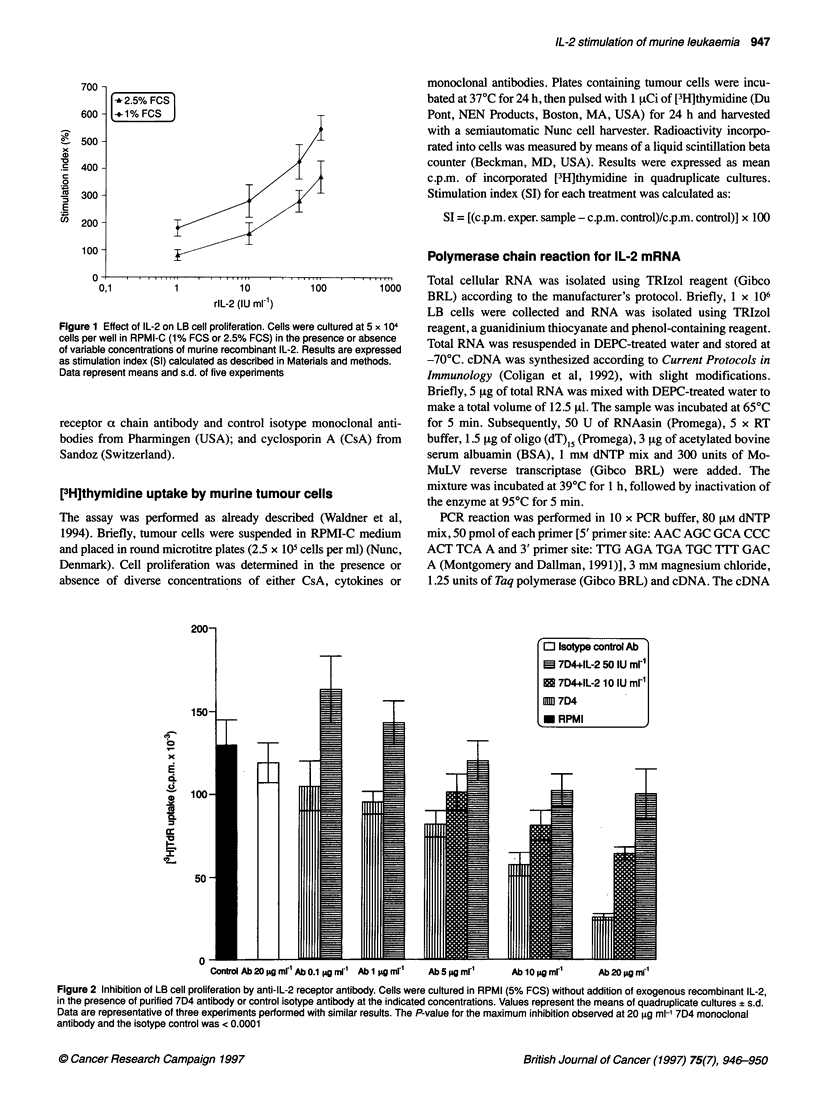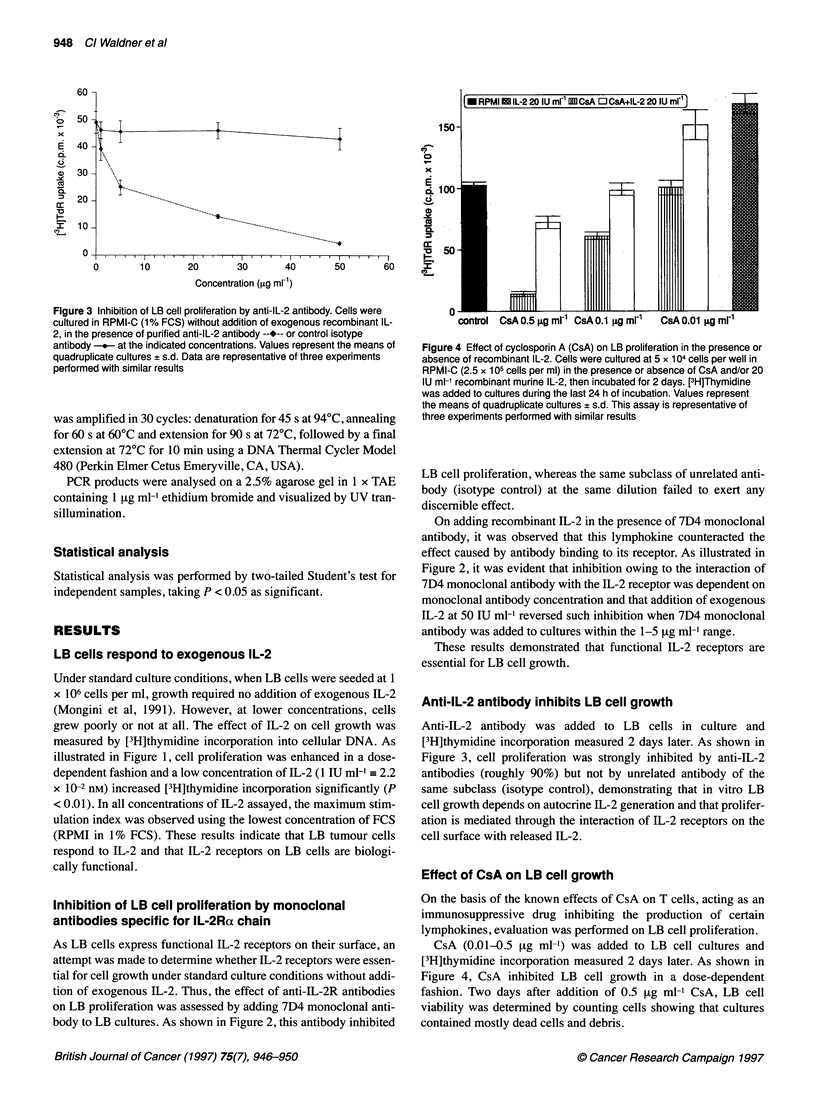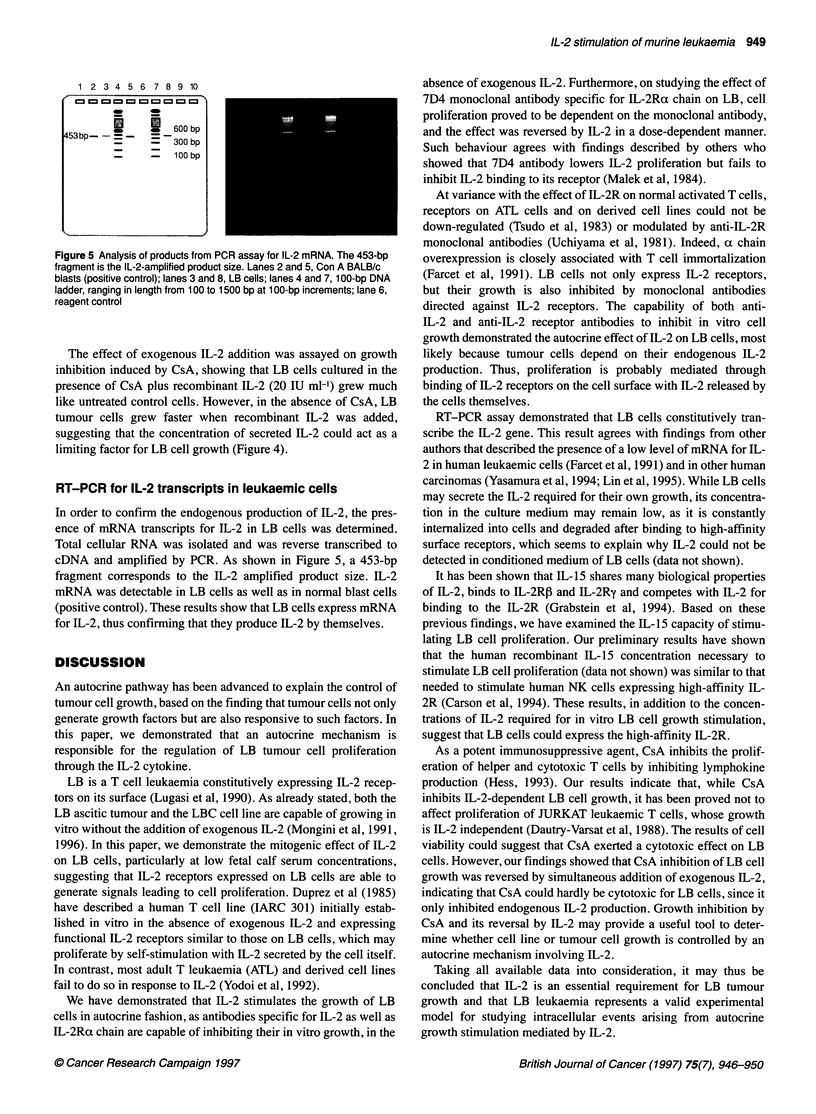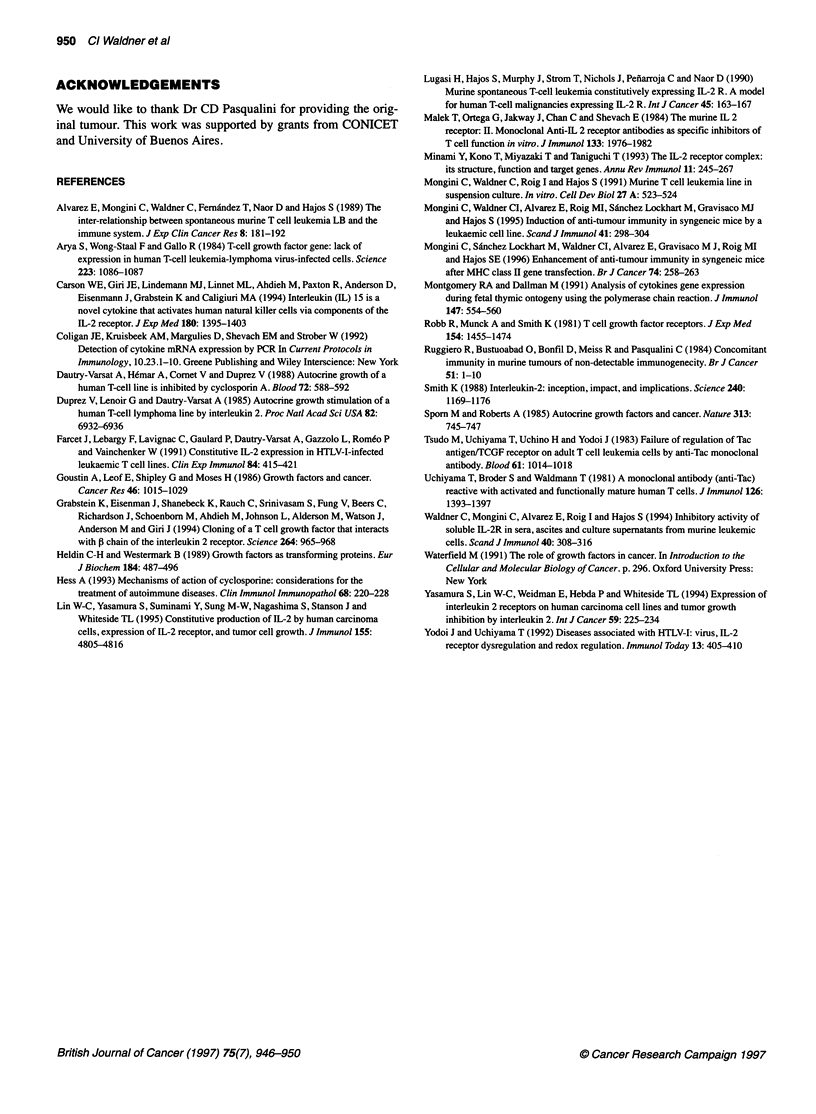# Interleukin 2 exerts autocrine stimulation on murine T-cell leukaemia growth.

**DOI:** 10.1038/bjc.1997.165

**Published:** 1997

**Authors:** C. I. Waldner, C. Mongini, E. Alvarez, M. SÃ¡nchez Lockhart, M. J. Gravisaco, S. E. Hajos

**Affiliations:** CÃ¡tedra de Immunologia, Facultad de Farmacia y BioquÃ­mica, Universidad de Buenos Aires, IDEHU, Argentina.

## Abstract

**Images:**


British Journal of Cancer (1997) 75(7), 946–950
© 1997 Cancer Research Campaign

### Interleukin 2 exerts autocrine stimulation on murine T-cell leukaemia growth

**CI Waldner, C Mongini, E Alvarez, M Sánchez Lockhart, MJ Gravisaco and SE Hajos**

Cátedra de Inmunología, Facultad de Farmacia y Bioquímica, Universidad de Buenos Aires, IDEHU: Instituto de Estudios de la Inmunidad Humoral, Argentina

**Summary** As it has been suggested that an autocrine mechanism may control tumour cell growth, in this work cells from a spontaneous murine T lymphocyte leukaemia (LB) expressing the interleukin-2 receptor (IL-2R) (CD25) were evaluated in vitro for IL-2-mediated autocrine growth. Cells grew readily in culture and proliferation was enhanced by the addition of recombinant IL-2 but inhibited by monoclonal antibodies against either IL-2 or IL-2 receptor, in the absence of exogenous IL-2. Cyclosporin A also inhibited LB cell growth. However, when exogenous IL-2 was added together with cyclosporin A, cell proliferation proved similar to controls. Using reverse transcription polymerase chain reaction (PCR), mRNA for IL-2 was found to be present in tumour cells. Our findings support the hypothesis that LB tumour cell proliferation is mediated by an autocrine pathway involving endogenous IL-2 generation, despite the fact that these cells are not dependent on exogenous IL-2 to grow in culture.

**Keywords:** autocrine growth; interleukin 2; murine leukaemia

Autocrine generation of growth factors has been advanced as a crucial component of tumorigenesis and autocrine secretion is considered to play a major role in malignant transformation (Sporn and Roberts, 1985; Goustin et al, 1986; Heldin and Westermark, 1989). A large number of studies have shown that growth factors are capable of mediating both positive and negative proliferative signals depending on the target cell (Waterfield, 1991). The release of immunomodulatory factors by neoplasms seems quite common, so that a variety of tumour-derived cytokines are liable to affect the immune response by diverse pathways.

IL-2 itself is released by T lymphocytes following stimulation by either antigen or mitogen and promotes long-term in vitro growth of normal T cells through specific surface receptors, which appear on activation (Robb et al, 1981; Minami et al, 1993) and are transiently expressed (Smith, 1988). Although certain human T cell leukaemia virus type I (HTLV-I) transformed cell lines established in vitro in the presence of IL-2 have become independent of this cytokine, they still respond to and synthesize IL-2 (Arya et al, 1984). However, relatively few HTLV-I-transformed cell lines behave likewise, suggesting that IL-2 may well stimulate their growth by an autocrine pathway.

Previously, we have studied a spontaneous murine T cell leukaemia, termed LB, which constitutively expresses the IL-2 receptor (CD25) (Lugasi et al, 1990), and we have also established a cell line, LBC, derived from the LB tumour (Mongini et al, 1991, 1995), which so far has not been grown in the presence of IL-2, and which bears IL-2 receptors on its cell surface similar to parent cells. In addition, in previous work we have demonstrated that LB cells release soluble IL-2R into the bloodstream and ascitic fluid or into conditioned medium, thus inhibiting in vitro growth of both normal splenocytes and tumour cells themselves (Waldner et al, 1994).

The present work was carried out to determine whether autocrine stimulation by IL-2 was involved in LB cell growth. Our results demonstrated that such growth was enhanced in response to exogenous IL-2 but impaired in the presence of either anti-IL-2 or anti-IL-2R monoclonal antibodies. In addition, mRNA for IL-2 was detected in LB cells. These results support the involvement of autocrine IL-2 stimulation in the growth of LB leukaemic cells.

### MATERIALS AND METHODS

### Mice

Normal BALB/c mice of either sex (2–4 months old), raised in the animal colony at the Facultad de Farmacia y Bioquímica, University of Buenos Aires, Argentina, and maintained on Cargill pellets and water *ad libitum*, were used.

### RPMI complete medium (RPMI-C)

RPMI-1640 (Gibco, Grand Island, NY, USA) was supplemented with 10% fetal calf serum (FCS), 2 mM L-glutamine, 20 mM Hepes buffer, 100 μg $ml^{-1}$ penicillin, 150 μg $ml^{-1}$ streptomycin and 50 μM 2-mercaptoethanol.

### Tumours

LB lymphoid leukaemia arose spontaneously in a 6 month-old BALB/c male mouse as a lymphocytic T-cell leukaemia (Ruggiero et al, 1984), and this tumour was maintained by serial passing in the peritoneal cavity of syngeneic hosts (Alvarez et al, 1989).

### Drugs, cytokines and monoclonal antibodies

Recombinant murine IL-2 and monoclonal anti-IL-2 antibody were purchased from Genzyme (USA); 7D4 monoclonal anti-IL-2

*Received 31 July 1996*
*Revised 16 October 1996*
*Accepted 22 October 1996*

*Correspondence to:* C Waldner, Cátedra de Inmunología, Facultad de Farmacia y Bioquímica, Junín 956 4° Piso, Buenos Aires 1113, Argentina

**Figure 1** Effect of IL-2 on LB cell proliferation. Cells were cultured at 5 × 10[4] cells per well in RPMI-C (1% FCS or 2.5% FCS) in the presence or absence of variable concentrations of murine recombinant IL-2. Results are expressed as stimulation index (SI) calculated as described in Materials and methods. Data represent means and s.d. of five experiments receptor α chain antibody and control isotype monoclonal antibodies from Pharmingen (USA); and cyclosporin A (CsA) from Sandoz (Switzerland).

### [$^3$H]thymidine uptake by murine tumour cells

The assay was performed as already described (Waldner et al, 1994). Briefly, tumour cells were suspended in RPMI-C medium and placed in round microtitre plates (2.5 × 10$^5$ cells per ml) (Nunc, Denmark). Cell proliferation was determined in the presence or absence of diverse concentrations of either CsA, cytokines or monoclonal antibodies. Plates containing tumour cells were incubated at 37°C for 24 h, then pulsed with 1 µCi of [$^3$H]thymidine (Du Pont, NEN Products, Boston, MA, USA) for 24 h and harvested with a semiautomatic Nunc cell harvester. Radioactivity incorporated into cells was measured by means of a liquid scintillation beta counter (Beckman, MD, USA). Results were expressed as mean c.p.m. of incorporated [$^3$H]thymidine in quadruplicate cultures. Stimulation index (SI) for each treatment was calculated as:

$$SI = [(\text{c.p.m. exper. sample} - \text{c.p.m. control})/\text{c.p.m. control})] \times 100$$

### Polymerase chain reaction for IL-2 mRNA

Total cellular RNA was isolated using TRIzol reagent (Gibco BRL) according to the manufacturer's protocol. Briefly, 1 × 10$^6$ LB cells were collected and RNA was isolated using TRIzol reagent, a guanidinium thiocyanate and phenol-containing reagent. Total RNA was resuspended in DEPC-treated water and stored at –70°C. cDNA was synthesized according to *Current Protocols in Immunology* (Coligan et al, 1992), with slight modifications. Briefly, 5 µg of total RNA was mixed with DEPC-treated water to make a total volume of 12.5 µl. The sample was incubated at 65°C for 5 min. Subsequently, 50 U of RNAasin (Promega), 5 × RT buffer, 1.5 µg of oligo (dT)$_{15}$ (Promega), 3 µg of acetylated bovine serum albuamin (BSA), 1 mM dNTP mix and 300 units of Mo-MuLV reverse transcriptase (Gibco BRL) were added. The mixture was incubated at 39°C for 1 h, followed by inactivation of the enzyme at 95°C for 5 min.

PCR reaction was performed in 10 × PCR buffer, 80 µM dNTP mix, 50 pmol of each primer [5′ primer site: AAC AGC GCA CCC ACT TCA A and 3′ primer site: TTG AGA TGA TGC TTT GAC A (Montgomery and Dallman, 1991)], 3 mM magnesium chloride, 1.25 units of *Taq* polymerase (Gibco BRL) and cDNA. The cDNA

**Figure 2** Inhibition of LB cell proliferation by anti-IL-2 receptor antibody. Cells were cultured in RPMI (5% FCS) without addition of exogenous recombinant IL-2, in the presence of purified 7D4 antibody or control isotype antibody at the indicated concentrations. Values represent the means of quadruplicate cultures ± s.d. Data are representative of three experiments performed with similar results. The *P*-value for the maximum inhibition observed at 20 µg ml$^{-1}$ 7D4 monoclonal antibody and the isotype control was < 0.0001

**Figure 3** Inhibition of LB cell proliferation by anti-IL-2 antibody. Cells were cultured in RPMI-C (1% FCS) without addition of exogenous recombinant IL-2, in the presence of purified anti-IL-2 antibody --◆-- or control isotype antibody —◆— at the indicated concentrations. Values represent the means of quadruplicate cultures ± s.d. Data are representative of three experiments performed with similar results

**Figure 4** Effect of cyclosporin A (CsA) on LB proliferation in the presence or absence of recombinant IL-2. Cells were cultured at 5 × $10^4$ cells per well in RPMI-C (2.5 × $10^5$ cells per ml) in the presence or absence of CsA and/or 20 IU $ml^{-1}$ recombinant murine IL-2, then incubated for 2 days. [$^3$H]Thymidine was added to cultures during the last 24 h of incubation. Values represent the means of quadruplicate cultures ± s.d. This assay is representative of three experiments performed with similar results was amplified in 30 cycles: denaturation for 45 s at 94°C, annealing for 60 s at 60°C and extension for 90 s at 72°C, followed by a final extension at 72°C for 10 min using a DNA Thermal Cycler Model 480 (Perkin Elmer Cetus Emeryville, CA, USA).

PCR products were analysed on a 2.5% agarose gel in 1 × TAE containing 1 μg $ml^{-1}$ ethidium bromide and visualized by UV transillumination.

### Statistical analysis

Statistical analysis was performed by two-tailed Student's test for independent samples, taking $P < 0.05$ as significant.

### RESULTS

### LB cells respond to exogenous IL-2

Under standard culture conditions, when LB cells were seeded at 1 × $10^6$ cells per ml, growth required no addition of exogenous IL-2 (Mongini et al, 1991). However, at lower concentrations, cells grew poorly or not at all. The effect of IL-2 on cell growth was measured by [$^3$H]thymidine incorporation into cellular DNA. As illustrated in Figure 1, cell proliferation was enhanced in a dose-dependent fashion and a low concentration of IL-2 (1 IU $ml^{-1}$ ≡ 2.2 × $10^{-2}$ nM) increased [$^3$H]thymidine incorporation significantly ($P < 0.01$). In all concentrations of IL-2 assayed, the maximum stimulation index was observed using the lowest concentration of FCS (RPMI in 1% FCS). These results indicate that LB tumour cells respond to IL-2 and that IL-2 receptors on LB cells are biologically functional.

### Inhibition of LB cell proliferation by monoclonal antibodies specific for IL-2Rα chain

As LB cells express functional IL-2 receptors on their surface, an attempt was made to determine whether IL-2 receptors were essential for cell growth under standard culture conditions without addition of exogenous IL-2. Thus, the effect of anti-IL-2R antibodies on LB proliferation was assessed by adding 7D4 monoclonal antibody to LB cultures. As shown in Figure 2, this antibody inhibited LB cell proliferation, whereas the same subclass of unrelated antibody (isotype control) at the same dilution failed to exert any discernible effect.

On adding recombinant IL-2 in the presence of 7D4 monoclonal antibody, it was observed that this lymphokine counteracted the effect caused by antibody binding to its receptor. As illustrated in Figure 2, it was evident that inhibition owing to the interaction of 7D4 monoclonal antibody with the IL-2 receptor was dependent on monoclonal antibody concentration and that addition of exogenous IL-2 at 50 IU $ml^{-1}$ reversed such inhibition when 7D4 monoclonal antibody was added to cultures within the 1–5 μg $ml^{-1}$ range.

These results demonstrated that functional IL-2 receptors are essential for LB cell growth.

### Anti-IL-2 antibody inhibits LB cell growth

Anti-IL-2 antibody was added to LB cells in culture and [$^3$H]thymidine incorporation measured 2 days later. As shown in Figure 3, cell proliferation was strongly inhibited by anti-IL-2 antibodies (roughly 90%) but not by unrelated antibody of the same subclass (isotype control), demonstrating that in vitro LB cell growth depends on autocrine IL-2 generation and that proliferation is mediated through the interaction of IL-2 receptors on the cell surface with released IL-2.

### Effect of CsA on LB cell growth

On the basis of the known effects of CsA on T cells, acting as an immunosuppressive drug inhibiting the production of certain lymphokines, evaluation was performed on LB cell proliferation.

CsA (0.01–0.5 μg $ml^{-1}$) was added to LB cell cultures and [$^3$H]thymidine incorporation measured 2 days later. As shown in Figure 4, CsA inhibited LB cell growth in a dose-dependent fashion. Two days after addition of 0.5 μg $ml^{-1}$ CsA, LB cell viability was determined by counting cells showing that cultures contained mostly dead cells and debris.

© Cancer Research Campaign 1997

**Figure 5** Analysis of products from PCR assay for IL-2 mRNA. The 453-bp fragment is the IL-2-amplified product size. Lanes 2 and 5, Con A BALB/c blasts (positive control); lanes 3 and 8, LB cells; lanes 4 and 7, 100-bp DNA ladder, ranging in length from 100 to 1500 bp at 100-bp increments; lane 6, reagent control

The effect of exogenous IL-2 addition was assayed on growth inhibition induced by CsA, showing that LB cells cultured in the presence of CsA plus recombinant IL-2 (20 IU $ml^{-1}$) grew much like untreated control cells. However, in the absence of CsA, LB tumour cells grew faster when recombinant IL-2 was added, suggesting that the concentration of secreted IL-2 could act as a limiting factor for LB cell growth (Figure 4).

### RT–PCR for IL-2 transcripts in leukaemic cells

In order to confirm the endogenous production of IL-2, the presence of mRNA transcripts for IL-2 in LB cells was determined. Total cellular RNA was isolated and was reverse transcribed to cDNA and amplified by PCR. As shown in Figure 5, a 453-bp fragment corresponds to the IL-2 amplified product size. IL-2 mRNA was detectable in LB cells as well as in normal blast cells (positive control). These results show that LB cells express mRNA for IL-2, thus confirming that they produce IL-2 by themselves.

### DISCUSSION

An autocrine pathway has been advanced to explain the control of tumour cell growth, based on the finding that tumour cells not only generate growth factors but are also responsive to such factors. In this paper, we demonstrated that an autocrine mechanism is responsible for the regulation of LB tumour cell proliferation through the IL-2 cytokine.

LB is a T cell leukaemia constitutively expressing IL-2 receptors on its surface (Lugasi et al, 1990). As already stated, both the LB ascitic tumour and the LBC cell line are capable of growing in vitro without the addition of exogenous IL-2 (Mongini et al, 1991, 1996). In this paper, we demonstrate the mitogenic effect of IL-2 on LB cells, particularly at low fetal calf serum concentrations, suggesting that IL-2 receptors expressed on LB cells are able to generate signals leading to cell proliferation. Duprez et al (1985) have described a human T cell line (IARC 301) initially established in vitro in the absence of exogenous IL-2 and expressing functional IL-2 receptors similar to those on LB cells, which may proliferate by self-stimulation with IL-2 secreted by the cell itself. In contrast, most adult T leukaemia (ATL) and derived cell lines fail to do so in response to IL-2 (Yodoi et al, 1992).

We have demonstrated that IL-2 stimulates the growth of LB cells in autocrine fashion, as antibodies specific for IL-2 as well as IL-2Rα chain are capable of inhibiting their in vitro growth, in the absence of exogenous IL-2. Furthermore, on studying the effect of 7D4 monoclonal antibody specific for IL-2Rα chain on LB, cell proliferation proved to be dependent on the monoclonal antibody, and the effect was reversed by IL-2 in a dose-dependent manner. Such behaviour agrees with findings described by others who showed that 7D4 antibody lowers IL-2 proliferation but fails to inhibit IL-2 binding to its receptor (Malek et al, 1984).

At variance with the effect of IL-2R on normal activated T cells, receptors on ATL cells and on derived cell lines could not be down-regulated (Tsudo et al, 1983) or modulated by anti-IL-2R monoclonal antibodies (Uchiyama et al, 1981). Indeed, α chain overexpression is closely associated with T cell immortalization (Farcet et al, 1991). LB cells not only express IL-2 receptors, but their growth is also inhibited by monoclonal antibodies directed against IL-2 receptors. The capability of both anti-IL-2 and anti-IL-2 receptor antibodies to inhibit in vitro cell growth demonstrated the autocrine effect of IL-2 on LB cells, most likely because tumour cells depend on their endogenous IL-2 production. Thus, proliferation is probably mediated through binding of IL-2 receptors on the cell surface with IL-2 released by the cells themselves.

RT–PCR assay demonstrated that LB cells constitutively transcribe the IL-2 gene. This result agrees with findings from other authors that described the presence of a low level of mRNA for IL-2 in human leukaemic cells (Farcet et al, 1991) and in other human carcinomas (Yasamura et al, 1994; Lin et al, 1995). While LB cells may secrete the IL-2 required for their own growth, its concentration in the culture medium may remain low, as it is constantly internalized into cells and degraded after binding to high-affinity surface receptors, which seems to explain why IL-2 could not be detected in conditioned medium of LB cells (data not shown).

It has been shown that IL-15 shares many biological properties of IL-2, binds to IL-2Rβ and IL-2Rγ and competes with IL-2 for binding to the IL-2R (Grabstein et al, 1994). Based on these previous findings, we have examined the IL-15 capacity of stimulating LB cell proliferation. Our preliminary results have shown that the human recombinant IL-15 concentration necessary to stimulate LB cell proliferation (data not shown) was similar to that needed to stimulate human NK cells expressing high-affinity IL-2R (Carson et al, 1994). These results, in addition to the concentrations of IL-2 required for in vitro LB cell growth stimulation, suggest that LB cells could express the high-affinity IL-2R.

As a potent immunosuppressive agent, CsA inhibits the proliferation of helper and cytotoxic T cells by inhibiting lymphokine production (Hess, 1993). Our results indicate that, while CsA inhibits IL-2-dependent LB cell growth, it has been proved not to affect proliferation of JURKAT leukaemic T cells, whose growth is IL-2 independent (Dautry-Varsat et al, 1988). The results of cell viability could suggest that CsA exerted a cytotoxic effect on LB cells. However, our findings showed that CsA inhibition of LB cell growth was reversed by simultaneous addition of exogenous IL-2, indicating that CsA could hardly be cytotoxic for LB cells, since it only inhibited endogenous IL-2 production. Growth inhibition by CsA and its reversal by IL-2 may provide a useful tool to determine whether cell line or tumour cell growth is controlled by an autocrine mechanism involving IL-2.

Taking all available data into consideration, it may thus be concluded that IL-2 is an essential requirement for LB tumour growth and that LB leukaemia represents a valid experimental model for studying intracellular events arising from autocrine growth stimulation mediated by IL-2.

*© Cancer Research Campaign 1997*

### ACKNOWLEDGEMENTS

We would like to thank Dr CD Pasqualini for providing the original tumour. This work was supported by grants from CONICET and University of Buenos Aires.

### REFERENCES

Alvarez E, Mongini C, Waldner C, Fernández T, Naor D and Hajos S (1989) The inter-relationship between spontaneous murine T cell leukemia LB and the immune system. *J Exp Clin Cancer Res* **8**: 181–192

Arya S, Wong-Staal F and Gallo R (1984) T-cell growth factor gene: lack of expression in human T-cell leukemia-lymphoma virus-infected cells. *Science* **223**: 1086–1087

Carson WE, Giri JE, Lindemann MJ, Linnet ML, Ahdieh M, Paxton R, Anderson D, Eisenmann J, Grabstein K and Caligiuri MA (1994) Interleukin (IL) 15 is a novel cytokine that activates human natural killer cells via components of the IL-2 receptor. *J Exp Med* **180**: 1395–1403

Coligan JE, Kruisbeek AM, Margulies D, Shevach EM and Strober W (1992) Detection of cytokine mRNA expression by PCR In *Current Protocols in Immunology*, 10.23.1–10. Greene Publishing and Wiley Interscience: New York

Dautry-Varsat A, Hémar A, Cornet V and Duprez V (1988) Autocrine growth of a human T-cell line is inhibited by cyclosporin A. *Blood* **72**: 588–592

Duprez V, Lenoir G and Dautry-Varsat A (1985) Autocrine growth stimulation of a human T-cell lymphoma line by interleukin 2. *Proc Natl Acad Sci USA* **82**: 6932–6936

Farcet J, Lebargy F, Lavignac C, Gaulard P, Dautry-Varsat A, Gazzolo L, Roméo P and Vainchenker W (1991) Constitutive IL-2 expression in HTLV-I-infected leukaemic T cell lines. *Clin Exp Immunol* **84**: 415–421

Goustin A, Leof E, Shipley G and Moses H (1986) Growth factors and cancer. *Cancer Res* **46**: 1015–1029

Grabstein K, Eisenman J, Shanebeck K, Rauch C, Srinivasam S, Fung V, Beers C, Richardson J, Schoenborn M, Ahdieh M, Johnson L, Alderson M, Watson J, Anderson M and Giri J (1994) Cloning of a T cell growth factor that interacts with β chain of the interleukin 2 receptor. *Science* **264**: 965–968

Heldin C-H and Westermark B (1989) Growth factors as transforming proteins. *Eur J Biochem* **184**: 487–496

Hess A (1993) Mechanisms of action of cyclosporine: considerations for the treatment of autoimmune diseases. *Clin Immunol Immunopathol* **68**: 220–228

Lin W-C, Yasamura S, Suminami Y, Sung M-W, Nagashima S, Stanson J and Whiteside TL (1995) Constitutive production of IL-2 by human carcinoma cells, expression of IL-2 receptor, and tumor cell growth. *J Immunol* **155**: 4805–4816

Lugasi H, Hajos S, Murphy J, Strom T, Nichols J, Peñarroja C and Naor D (1990) Murine spontaneous T-cell leukemia constitutively expressing IL-2 R. A model for human T-cell malignancies expressing IL-2 R. *Int J Cancer* **45**: 163–167

Malek T, Ortega G, Jakway J, Chan C and Shevach E (1984) The murine IL 2 receptor: II. Monoclonal Anti-IL 2 receptor antibodies as specific inhibitors of T cell function *in vitro*. *J Immunol* **133**: 1976–1982

Minami Y, Kono T, Miyazaki T and Taniguchi T (1993) The IL-2 receptor complex: its structure, function and target genes. *Annu Rev Immunol* **11**: 245–267

Mongini C, Waldner C, Roig I and Hajos S (1991) Murine T cell leukemia line in suspension culture. *In vitro. Cell Dev Biol* **27 A**: 523–524

Mongini C, Waldner CI, Alvarez E, Roig MI, Sánchez Lockhart M, Gravisaco MJ and Hajos S (1995) Induction of anti-tumour immunity in syngeneic mice by a leukaemic cell line. *Scand J Immunol* **41**: 298–304

Mongini C, Sánchez Lockhart M, Waldner CI, Alvarez E, Gravisaco M J, Roig MI and Hajos SE (1996) Enhancement of anti-tumour immunity in syngeneic mice after MHC class II gene transfection. *Br J Cancer* **74**: 258–263

Montgomery RA and Dallman M (1991) Analysis of cytokines gene expression during fetal thymic ontogeny using the polymerase chain reaction. *J Immunol* **147**: 554–560

Robb R, Munck A and Smith K (1981) T cell growth factor receptors. *J Exp Med* **154**: 1455–1474

Ruggiero R, Bustuoabad O, Bonfil D, Meiss R and Pasqualini C (1984) Concomitant immunity in murine tumours of non-detectable immunogenecity. *Br J Cancer* **51**: 1–10

Smith K (1988) Interleukin-2: inception, impact, and implications. *Science* **240**: 1169–1176

Sporn M and Roberts A (1985) Autocrine growth factors and cancer. *Nature* **313**: 745–747

Tsudo M, Uchiyama T, Uchino H and Yodoi J (1983) Failure of regulation of Tac antigen/TCGF receptor on adult T cell leukemia cells by anti-Tac monoclonal antibody. *Blood* **61**: 1014–1018

Uchiyama T, Broder S and Waldmann T (1981) A monoclonal antibody (anti-Tac) reactive with activated and functionally mature human T cells. *J Immunol* **126**: 1393–1397

Waldner C, Mongini C, Alvarez E, Roig I and Hajos S (1994) Inhibitory activity of soluble IL-2R in sera, ascites and culture supernatants from murine leukemic cells. *Scand J Immunol* **40**: 308–316

Waterfield M (1991) The role of growth factors in cancer. In *Introduction to the Cellular and Molecular Biology of Cancer*. p. 296. Oxford University Press: New York

Yasamura S, Lin W-C, Weidman E, Hebda P and Whiteside TL (1994) Expression of interleukin 2 receptors on human carcinoma cell lines and tumor growth inhibition by interleukin 2. *Int J Cancer* **59**: 225–234

Yodoi J and Uchiyama T (1992) Diseases associated with HTLV-I: virus, IL-2 receptor dysregulation and redox regulation. *Immunol Today* **13**: 405–410

© Cancer Research Campaign 1997